# Immunohistochemical analysis using cell block technique leads accurate diagnosis of ovarian malignant lymphoma: A case report

**DOI:** 10.1016/j.ijscr.2020.03.013

**Published:** 2020-03-14

**Authors:** Kosuke Hiramatsu, Kaoru Fukui, Ikuko Sawada, Kentaro Kuritani, Masafumi Takahashi, Tomoko Kanayama, Hiromi Ugaki, Mirang Kim, Megumu Inoue, Hayato Kimura, Kyoka Amemiya

**Affiliations:** a Department of Obstetrics and Gynecology, Itami City Hospital, Japan; b Department of Hematology, Itami City Hospital, Japan; c Department of Pathology, Itami City Hospital, Japan

**Keywords:** Laparoscopic surgery, Ovarian cancer, Cell block, Lymphoma

## Abstract

**Introduction:**

Ovarian malignant lymphoma is a rare gynecologic disease and some patients show marked ascites, similar to that observed in advanced ovarian cancer. Although radical surgery improves prognosis of ovarian cancer, treatment of lymphoma is based on chemotherapy, therefore, differential diagnosis is crucial.

**Presentation of case:**

A 65-year-old woman presented with a 1-month history of abdominal distention. Pelvic ultrasonography showed an 11-cm solid mass in the pelvis. Computed tomography and magnetic resonance imaging revealed bilateral (mainly left) ovarian masses in the pelvis and multiple metastases. Laboratory examination revealed that serum CA125 levels were elevated, suggesting the existence of advanced ovarian cancer. To confirm the diagnosis, the ascites was removed via abdominocentesis. Although no malignant epithelial cells were observed, atypical lymphoid cells dispersed in the ascites were detected in the cytological analyses. Thus, for accurate diagnosis, we performed re-abdominocentesis and immunohistochemical (IHC) analysis using cell block technique. Cell block analysis showed negative staining for CD3 and positive staining for CD20 in large atypical lymphoid cells, suggesting the existence of large B-cell lymphoma. Repeat blood examination showed that the serum sIL-2R level was elevated. We decided to perform biopsy to make the final treatment decision. Histologically, the tumor demonstrated diffuse proliferation of large atypical lymphoid cells. IHC analysis showed CD3(-), CD5(+), and CD20(+). In addition, IHC analysis also showed CD79a(+), CD10(-), bcl-2(+), and cyclin D1(-). The final diagnosis was diffuse large B-cell lymphoma.

**Discussion and conclusion:**

Here, we present the case of a patient with ovarian malignant lymphoma that was diagnosed using cell block analysis.

## Highlights

• Treatment of ovarian malignant lymphoma is based on chemotherapy, although radical surgery improves prognosis of epithelial ovarian cancer.
• Immunohistochemical analysis using cell block technology helped us establish an accurate diagnosis of ovarian lymphoma.
• Abdominocentesis followed by cell block analysis is very useful for identifying ovarian malignancy before radical invasive operation.

## 1 Introduction

Ovarian malignant lymphoma is a rare gynecologic disease and some patients show marked ascites, similar to that observed in advanced ovarian cancer. Although radical surgery improves prognosis of ovarian cancer, treatment of lymphoma is based on chemotherapy, therefore, differential diagnosis is crucial.

**Fig. 1:**
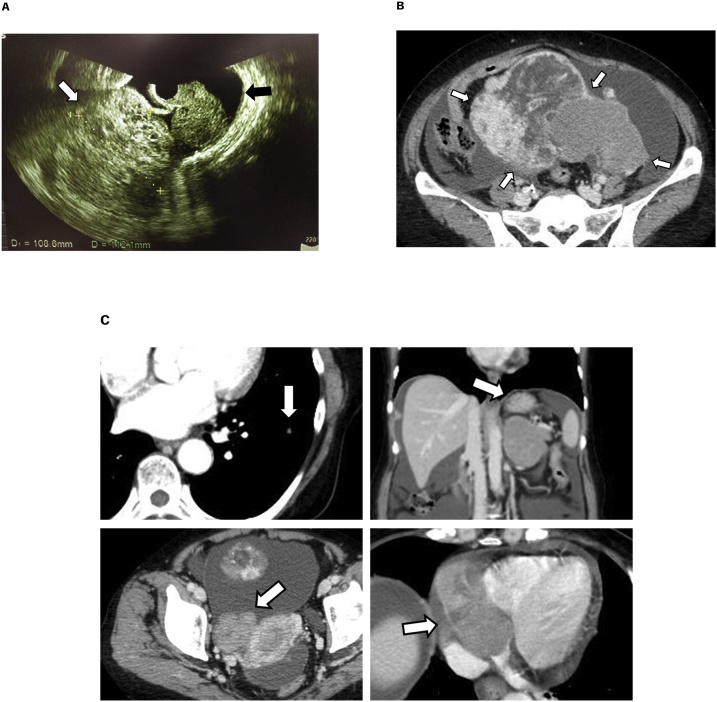
**A,** Pelvic ultrasonography showed an 11-cm solid mass in the pelvis that was rough, hard, and had poor mobility (white arrow). In addition, abundant ascites was also identified (black arrow). **B,** Computed tomography (CT) revealed a huge ovarian mass at left adnexa that occupied the pelvic cavity. **C,** CT also identified multiple metastases that included the lung (upper left), adrenal gland (upper right), right adnexa (lower left), and heart (lower right) (white arrow).

Cytological analysis based on the observation of ascites is very common in the initial diagnosis of advanced EOC. However, this method is limited in terms of the sensitivity. Therefore, cell block analysis is sometimes used to establish a pathological diagnosis (Fig. 1).

**Fig. 2:**
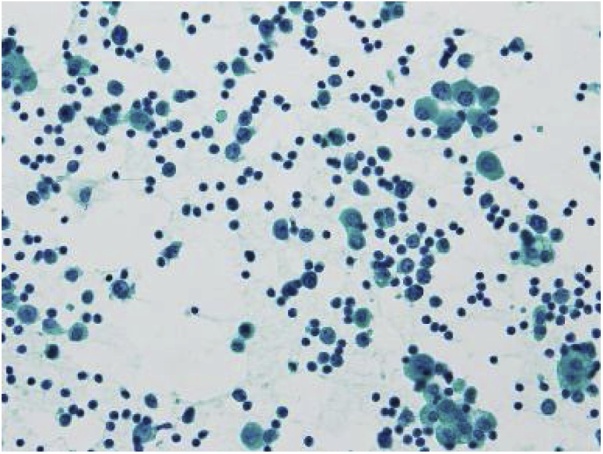
Although no malignant epithelial cells were observed, atypical lymphoid cells were dispersed in the ascites.

Here, we present the case of a patient with ovarian malignant lymphoma that was diagnosed using cell block analysis (Fig. 2).

This work has been reported in line with the SCARE criteria (Agha) [1].

## 2 Presentation of case

**Fig. 3:**
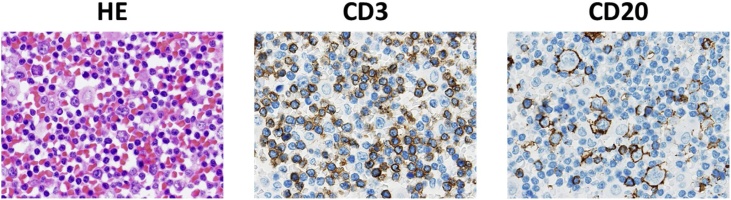
Cell block analysis showed negative staining of CD3 and positive staining of CD20 in large lymphocytes that were identified using hematoxylin-eosin stain.

A 65-year-old woman presented with a 1-month history of abdominal distention. Pelvic ultrasonography showed an 11-cm solid mass in the pelvis. Computed tomography and magnetic resonance imaging revealed bilateral (mainly left) ovarian masses in the pelvis and multiple metastases. Laboratory examination revealed that serum CA125 levels were elevated up to 1005.7 U/mL, suggesting the existence of advanced ovarian cancer. To confirm the diagnosis, the ascites was removed via abdominocentesis. Although no malignant epithelial cells were observed, atypical lymphoid cells dispersed in the ascites were detected in the cytological analyses. Thus, for accurate diagnosis, we performed re-abdominocentesis and immunohistochemical (IHC) analysis using cell block technique. Cell block analysis showed negative staining for CD3 and positive staining for CD20 in large atypical lymphoid cells identified using hematoxylin-eosin stain (Fig. 3), suggesting the existence of large B-cell lymphoma.

**Fig. 4:**
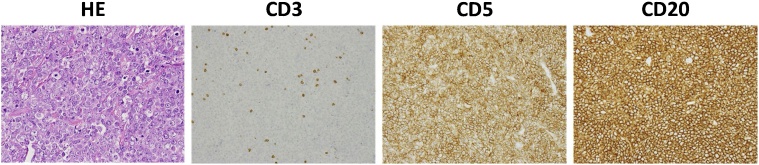
Immunohistochemical analysis using biopsy fragment showed negative staining of CD3 and positive staining of CD5 and C20.

Repeat blood examination showed that the serum sIL-2R level was 3657 U/mL. After discussion with the hematologist, we decided to perform biopsy to make the final treatment decision. Laparoscopic operation revealed a 20-cm, irregular, poorly movable mass in the pelvis. We tried to obtain a sample of the surface of this tumor; however, a lot of bleeding was expected, and there was no suitable metastatic cite for sampling on the peritoneal surface. Thus, we changed the operation method to laparotomy and resected 1 cm of the tissue. After resection, we used TachoComb to stop the bleeding. Histologically, the tumor demonstrated diffuse proliferation of large atypical lymphoid cells and a high mitotic rate. IHC analysis showed CD3(-), CD5(+), and CD20(+). In addition, IHC analysis also showed CD79a(+), CD10(-), bcl-2(+), and cyclin D1(-) (Fig. 4). The final diagnosis was diffuse large B-cell lymphoma (DLBCL) (Cotsworlds-modified Ann Arbor classification, stage IV).

After the operation, the patient was treated with the R-CHOP regimen (rituximab, cyclophosphamide, doxorubicin, vincristine, and prednisolone). The tumor volume and markers decreased substantially; however, she relapsed soon. She died on postoperative day 191.

## 3 Discussion

In this case, IHC analysis using cell block technology helped us establish an accurate diagnosis of ovarian DLBCL, and we could avoid aggressive cytoreductive surgery that is applied for EOC and is unsuitable for DLBCL patients. To our knowledge, this is the first report wherein cell block analysis using ascites aided the diagnosis of DLBCL.

Over 70% of EOC patients are diagnosed with advanced stage (Zheng) [2]; in such patients with ascites fluid, cytological analysis is usually performed for the initial diagnosis. Abdominocentesis followed by cytological analysis of ascites is widely used easily and safely; however, in some cases, it is difficult to distinguish malignant cells from reactive mesothelial cells. Therefore, the sensitivity of cytological analysis of ascites is only 40%–70% (Dey) [3]. In fact, we hypothesized the existence of advanced EOC with abundant ascites and multiple metastases; however, it was impossible to detect epithelial malignant cells via cytological analysis of ascites. Thus, we performed cell block analysis on the ascites. The sensitivity of cell block analysis to detect malignancy is 80%–100% (Dey) [4] (Matreja) [5] (Shivakumarswamy) [3]. Compared to cytological analysis, cell block analysis offers the advantage of developing multiple sectioning of paraffin-embedded cells, observing structural patterns, and performing IHC analysis. This technique also can be applied for fluorescence in situ hybridization, electron microscopic analysis, and genomic analysis. Fluid samples contain not only malignant cells but also non-malignant cells including inflammatory and mesothelial cells, thus, obtaining more ascites is important for that, especially when cytological analysis shows no malignancy, but malignancy is suspected clinically.

In our case, IHC analysis using cell block technique showed the possibility of ovarian lymphoma for the first time, and subsequent blood examination also suggested the existence of lymphoma. Primary ovarian lymphoma and lymphoma with ovarian metastasis are rarely observed; therefore, imaging diagnosis could lead to an inaccurate diagnosis. In fact, patients with ovarian lymphoma who were treated for EOC and were diagnosed with lymphoma after invasive radical operation owing to the challenges faced in the differential diagnosis (Chien) [6] (Senol) [7] were reported. For EOC patients, the combination of cytoreductive radical surgery and platinum-based chemotherapy was performed. However, for DLBCL patients, treatment based on the R-CHOP regimen is usually performed. It is noteworthy that cytoreductive surgery does not improve the outcomes in patients with lymphoma (Senol) [7]. Therefore, accurate diagnosis before treatment is extremely important. In this case, after cell block analysis, we performed tumor tissue biopsy for careful management with the assistance of a pathologist and hematologist. The reason is that lymphocytes responding to inflammation resemble malignant lymphoma cells in some cases, and the analysis of distribution and structure of lymphoma cells in the tissue is also important for diagnosis. Moreover, fresh tissue sample enables to analyze cell surface antigen and specific chromosomal/genetic abnormalities in each case. We tried to perform the biopsy using laparoscopy, however, during the operation we determined to change to laparotomy. In general, laparoscopic biopsy is less invasive, thus, we could avoid radical invasive surgery. Abdominocentesis followed by cell block analysis could be considered a useful, minimally invasive and low-cost diagnostic method for ovarian malignancy with ascites.

For diagnosis of DLCBL, IHC analysis of CD3 and CD20 expression are crucial. DLCBL commonly shows CD3 (-) and CD20 (+) expression pattern. In addition, analysis of CD5 expression is also important for predicting the prognosis. CD5 (+) DLCBL reportedly rises in older patients and shows many aggressive clinical features compared to patients with CD5 (-) DCLBL (Yamaguchi) [8] (Xu-Monette) [9]. In fact, our patient showed high expression of CD5 and poor prognosis.

Ovarian lymphoma is very rare and has no specific clinical features; thus, imaging diagnosis against this rare lymphoma is very challenging. Fox et al. proposed the following diagnostic criteria for primary ovarian lymphoma in 1988 (Fox) [10]:

1. At the time of diagnosis, the lymphoma is clinically confined to the ovary, and a full investigation fails to reveal evidence of lymphoma elsewhere. A lymphoma will still be considered primary if the spread has occurred to the immediately adjacent lymph nodes or if there has been direct infiltration in the adjacent structures.
2. The peripheral blood and bone marrow should not contain any abnormal cells.
3. If further lymphomatous lesions occur at sites remote from the ovary, at least several months should have elapsed between the appearance of the ovarian and extra ovarian lesions.

Our case is not applied for these criteria (This case was identified in advanced stage with extra ovarian metastasis); however, limitations of this criteria for the diagnosis of primary ovarian lymphoma are reported (Breda) [11] (Perlman) [12] (Paladugu) [13]. These criteria tend to exclude all advanced “primary” ovarian lymphomas. In addition, recent management against malignant disease has shifted from origin-based to genomic character-based treatment. Genomic character-based new criteria of ovarian lymphoma might be needed.

In conclusion, ovarian lymphoma is a very rare disease. In patients with ascites, abdominocentesis followed by cell block analysis is very useful for identifying ovarian malignancy before radical invasive operation.

## Declaration of Competing Interest

No conflict of interest.

## Sources of funding

No funding for our research.

## Ethical approval

This research was approved by Ethnical committee of Itami City Hospital (No. 863).

## Consent

We obtained written and signed consent to publish this case report from the patient’s family.

## Author contribution

Kosuke Hiramatsu: study concept or design, data collection, data analysis, writing the paper.

Kaoru Fukui: data collection.

Ikuko Sawada: data collection.

Kentaro Kuritani: data collection.

Masaaki Takahashi: study concept or design, data collection.

Tomoko Kanayama: data collection.

Hiromi Ugaki: writing the paper.

Mirang Kim: writing the paper.

Megumu Inoue: study concept or design, data collection.

Hayato Kimura: data collection, data analysis.

Kyoka Amemiya: study concept or design, writing the paper.

## Registration of research studies

This study is observation study.

## Guarantor

Kyoka Amemiya.

## Provenance and peer review

Not commissioned, externally peer-reviewed.
